# Dengue fever manifesting with tetany as the first presentation of primary hypoparathyroidism: a case report

**DOI:** 10.1186/s13104-018-3701-2

**Published:** 2018-08-14

**Authors:** Rakitha Higgoda, Kasun Lokuketagoda, Thuvarakan Poobalasingham, Venura Wedagedara, Dilshan Perera, Kanapathipillai Thirumavalavan

**Affiliations:** National Hospital of Sri Lanka, Colombo, Sri Lanka

**Keywords:** Hypoparathyroidism, Hypocalcemia, Tetany, Dengue, Basal ganglia calcifications

## Abstract

**Background:**

Primary hypoparathyroidism is associated with diverse variety of symptomatology of hypocalcemia including seizures and tetany. We report a case of previously undiagnosed asymptomatic primary hypoparathyroidism with extensive basal ganglia calcifications presenting for the first time with hypocalcemic tetany during acute dengue infection. Although hypocalcemia is known to occur in dengue infection symptomatic hypocalcemia is very infrequent.

**Case presentation:**

A 32 year old male with short stature who has undergone bilateral cataract surgery 2 years ago but who was otherwise healthy, presented with fever and generalized body aches of 3 days duration and carpal spasms/tetany occurring on the third day of the illness. He was diagnosed to have acute dengue fever along with severe hypocalcemia. Subsequent workup confirmed that the patient had primary hypoparathyroidism with extensive basal ganglia and cerebellar calcifications which was previously undiagnosed. His acute illness and hypocalcemia was managed successfully and was commenced on regular calcium supplementations to alleviate the hypocalcemic effects of his chronic illness.

**Conclusion:**

Clinical features of hypocalcemia may not commonly manifest up to the same degree of severity of hypocalcemia in primary hypoparathyroidism even till late adulthood but potential early clues such as short stature and premature cataract should be actively investigated. Worsening of already existing hypocalcemia during acute dengue fever led to the ultimate diagnosis of primary hypoparathyroidism in this patient which was lifesaving.

## Background

Primary hypoparathyroidism is a relatively rare endocrine disorder with estimated prevalence in the United States of 37 per 100,000 person-years [1]. Primary hypoparathyroidism is defined by a low concentration of parathyroid hormone (PTH) with a concomitant low calcium level. In the absence of adequate PTH activity, the ionized calcium concentration in the extracellular fluid falls below the reference range, which may be variably symptomatic. Permanent primary hypoparathyroidism may be congenital or acquired.

Clinically, hypoparathyroidism manifests predominantly as neuromuscular dysfunction caused by hypocalcemia. The symptomatology includes, paresthesias (involving fingertips, toes, perioral area), hyperirritability, personality disturbances, seizures, laryngospasms and muscle cramps [2]. Chronic hypocalcemia, observed in primary hypoparathyroidism, is sometimes associated with ocular cataracts, abnormal dentition and dry, puffy, coarse skin.

Primary hypoparathyroidism may also cause extrapyramidal choreoathetoid syndromes when associated basal ganglia calcifications are present. A literature review of the clinical presentations of basal ganglia calcifications revealed that there are diverse presentations, the most common being seizures, mental deterioration, and disorders of cerebellar or extra-pyramidal function. Movement disorders, chorea or parkinsonism are present in 20–30% of patients with basal ganglia calcification [3]. However, most of these patients may remain asymptomatic and therefore not diagnosed until adulthood [4].

In patients with autoimmune polyglandular syndrome, idiopathic hypoparathyroidism is associated with adrenal insufficiency and moniliasis.

Dengue fever on the other hand is prevalent in epidemic numbers in Sri Lanka at present. Dengue fever is a mosquito-borne tropical disease caused by the dengue virus and is a major cause of morbidity and mortality in tropical regions. Its manifestations may vary from a simple undifferentiated febrile illness to a severe clinical syndrome involving fluid leakage leading to shock. According to the epidemiology unit, ministry of health of Sri Lanka, during the first 7 months of the year 2017, 122,384 suspected dengue cases have been reported to the Epidemiology Unit from all over the island [5].

Interestingly, low blood calcium levels have been demonstrated in patients with dengue infection and it has been found that hypocalcemia is more pronounced in more severe forms of dengue infection [6]. The cause of hypocalcemia in dengue fever is likely to be multifactorial. Possible mechanisms include reduced Na^+^-K adenosine triphosphatase (ATPase) activity, reduced Ca^2+^-ATPase activity, acquired parathyroid hormone deficiency, renal one-alpha hydroxylase insufficiency, reduced dietary vitamin D intake, reduced dietary calcium intake and leakage of calcium to the third space [7]. The derangements of calcium homeostasis are likely to be associated with myocardial dysfunction and cardiac arrhythmias in dengue as suggested by in vitro studies [7]. But symptomatic hypocalcemia occurring in dengue fever is exceedingly infrequent and the few reported cases of hypocalcemic tetany in dengue infection belong to the pediatric age group [8].

We report a patient with previously undiagnosed asymptomatic primary hypoparathyroidism who experiences hypocalcemic tetany for the first time in his life during an episode of acute dengue infection.

## Case presentation

A 32 year old manual labourer who is not on long term treatment for any medical comorbidities, presented to our medical unit with fever, headache and arthralgia of 3 days duration. Towards the latter part of the third day he has noticed tetany/carpal spasms of both hands which brought him to the hospital. On inquiry he denied any perioral or fingertip paresthesia. He had no symptoms to suggest a respiratory, gastrointestinal or genitourinary focus of infection. There was no photo/phonophobia or history of seizures or muscle cramps.

He has undergone surgery for bilateral posterior subcapsular cataract at the age of 30 years for which the cause has not been previously evaluated. Although he had experienced several episodes of febrile illnesses during his lifetime, he has never experienced carpal spasms along with any febrile illness before. He did not give any past history of any neck or thyroid surgeries or irradiation. He was not on any long term medications and there was no significant history of familial illnesses.

On examination he was short with a height of 142 cm with normal upper and lower body proportions. He was febrile with a viral exanthem involving the upper trunk. He was not overtly anxious or hyperventilating. Notably he was having bilateral carpal spams with positive Chvostek sign. Trousseau sign was negative. His vitals including the blood pressure were stable. No other skeletal deformities such as scoliosis or short metacarpals were noted.

Cardiovascular, respiratory and abdominal examination revealed no abnormality. Fundal examination did not reveal papilloedema and there was no significant muscle weakness or hyper-reflexia on examination of the motor system.

With the ongoing dengue epidemic in Sri Lanka, dengue fever was suspected and an urgent full blood count and serum ionized calcium level were ordered on admission as the patient had carpal spasms.

The full blood count was suggestive of an ongoing viral illness with leucopenia (3.2 × 10^9^/l) and thrombocytopenia (105 × 10^9^/L) and thus he was tentatively diagnosed as a possible case of dengue fever. Serum ionized calcium level was 0.62 mmol/l, which was considerably below the reference level (1.05–1.30 mmol/L). Calcium was replaced urgently with intravenous 10% calcium gluconate which relieved the patient of his hypocalcemic symptoms. ECG obtained later did not show any rhythm abnormalities or hypocalcemic changes. He was given regular intravenous 10% calcium gluconate (six hourly) to keep the serum ionized calcium levels within normal limits during the acute illness and daily serum ionized calcium levels were monitored.

Further work up was done to evaluate his hypocalcaemia while the patient was monitored and managed as for dengue fever according to the national guidelines for dengue fever management. Twice daily full blood counts were carried out to monitor the course of dengue fever.

His baseline renal and liver functions tests were normal except for slightly raised aspartate aminotransferase (AST) (92 μ/l, range 10–40 μ/l) and alanine aminotransferase (ALT) (87 μ/l, range 7–56 μ/l) levels and was attributed to the ongoing viral illness, which later came down to the baseline normal levels. Serum magnesium (0.9 mmol/l, range 0.8–1.1 mmol/l), alkaline phosphatase (ALP) (109 U/l, range 100–360 U/l) and 25-hydroxy vitamin D level were within normal limits.

Serum inorganic phosphate level was slightly elevated (1.6 mmol/l, range 0.8–1.5 mmol/l) and at this point hypoparathyroidism or pseudohypoparathyroidism needed to be excluded and the intact PTH level was obtained which was significantly low/undetectable (< 2.5 pg/ml, range 14–72 pg/ml).

**Fig. 1:**
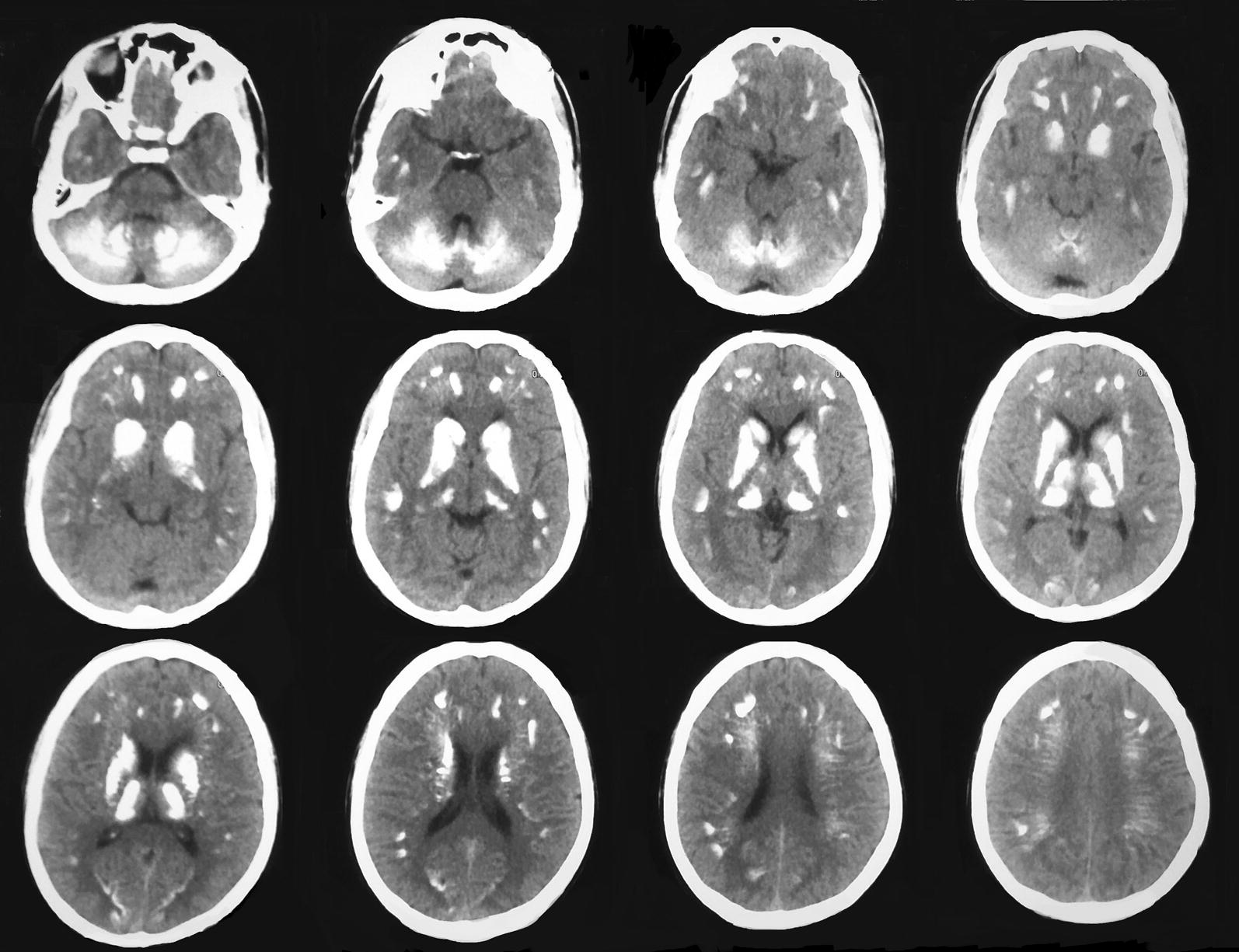
NCCT of brain. Bilateral symmetrical calcifications in cerebral subcortical regions, basal ganglia and cerebellum mainly involving biventral and semilunar lobules and posterior vermis

Non contrast computed tomography (NCCT) scan of the brain revealed multiple basal ganglia, subcortical and cerebellar calcifications (Fig. 1).

He did not demonstrate clinical or biochemical features of addison’s disease or mucocutaneous candidiasis to suggest that his hypoparathyroidism was part of an autoimmune polyglandular syndrome (Type 1) and there was no family history of conditions related to autoimmune polyglandular syndrome (Type 1). Nor did he demonstrate any specific syndromic features to suggest an underlying genetic disorder. Genetic analysis with regard to genetic hypoparathyroidism could not be carried out as facilities for such testing was not available in the country at the time of reporting.

Rest of the endocrine profile including thyroid stimulating hormone, free thyroxin, follicular stimulating hormone, luteinizing hormone and random 9 a.m. cortisol levels were normal.

His platelet count dropped to a minimum of 82 × 10^9^/l on day 5 of his febrile illness after which defervescence occurred with rising platelet and white blood cell counts over the next few days. He made an uneventful recovery without any features of dengue fluid leakage and by day 8 of his acute illness his platelet count rose up to 162 × 10^9^/l and had a normal serum ionized calcium level of 1.1 mmol/l. Dengue IgM/IgG serology done on day 8 of illness was positive which confirmed the initial diagnosis of dengue fever.

Following recovery from dengue fever he was started on regular oral calcium replacement therapy in the form of calcium carbonate along with one alfa cholecalciferol.

Patient was discharged from the hospital on oral calcium supplementation with the follow up plan of regular monitoring of serum calcium and 24 h urinary calcium excretion levels.

## Discussion and conclusions

Despite significantly low serum calcium levels seen in primary hypoparathyroidism its outward clinical manifestation might be a rare occurrence in some patients until late adulthood as described in the literature [8]. Our patient denied having any sort of hypocalcemic symptom until the current presentation with concurrent dengue fever. For the first time in his life, he experiences symptomatic hypocalcemia in the form of carpal spasms during this presentation with dengue fever. Interestingly, hypocalcemia has been also demonstrated in patients with dengue infection and according to the literature, hypocalcemia is more pronounced in more severe forms of dengue fever, particularly in dengue hemorrhagic fever (DHF) than in simple dengue fever (DF) for which the possible mechanisms were mentioned in the “Background” section [6]. However, the hypocalcemia occurring in dengue fever is rarely known to be symptomatic [7].

Although this particular patient had a milder form of dengue fever it can be assumed that worsening of his previously existing hypocalcemia during dengue infection led to its clinical manifestation. The fact that he has not experienced symptomatic hypocalcemia with any of the previous undifferentiated febrile illnesses, but with dengue infection, highlights the fact that hypocalcemia occurring in dengue is particularly profound as it led to worsening of already existing hypocalcemia which was manifested clinically as tetany. Hypocalcemia is known to cause negative physiological effects including embarrassment of cardiac function in dengue fever. Thus prompt identification and adequate correction of hypocalcemia of this patient who already had low baseline serum calcium levels was a very crucial point in management.

Although our patient did not have any symptomatic hypocalcemia previously, he was short in stature (142 cm) and had undergone bilateral cataract surgery at the age of 30 years, both of which are clues towards a possible underlying endocrinological disorder which have been unfortunately overlooked and further investigations had not been carried out previously.

Apart from above clinical features this particular patient also had extensive basal ganglia calcifications along with some subcortical and cerebellar calcifications as well. Surprisingly, this patient did not have any symptom of the possible diverse manifestations of basal ganglia calcifications mentioned afore in this article.

Primary hypoparathyroidism was confirmed by concomitant low intact PTH level with low serum calcium levels in this patient. The slightly high serum phosphate level was also supportive evidence. Hypomagnesemia and vitamin D deficiency were also excluded. There is always a risk of developing a seizure or a similar serious manifestation of hypocalcemia in a patient with untreated primary hypoparathyroidism at any point of life. Thus calcium replacement is considered lifesaving in a patient with a lifelong hypocalcemia. The goal of treatment in a patient with hypoparathyroidism is to maintain serum calcium in the low normal range in order to alleviate symptoms, but at the same time to avoid normocalcemia which may result in hypercalciuria. This can be achieved with cautious calcium and vitamin D supplementation along with monitoring of urinary calcium excretion [9].

We conclude that this patient is an interesting presentation of previously undiagnosed primary hypoparathyroidism presenting for the first time at the age of 32 years with hypocalcemic tetany precipitated by dengue infection which was treated successfully. It also highlights the point that hypoparathyroidism may remain unnoticed for years and potential clues like premature cataract should be extensively and vigilantly investigated.

## Abbreviations

PTH: parathyroid hormone
AST: aspartate aminotransferase
ALT: alanine aminotransferase
ALP: alkaline phosphatase
NCCT: non contrast computed tomography
DHF: dengue hemorrhagic fever
DF: dengue fever
